# Inappropriate use of medicines and associated factors in Brazil: an approach from a national household survey

**DOI:** 10.1093/heapol/czz038

**Published:** 2019-12-09

**Authors:** Vera Lucia Luiza, Luiz Villarinho Pereira Mendes, Noemia Urruth Leão Tavares, Andrea Damaso Bertoldi, Andréia Turmina Fontanella, Maria Auxiliadora Oliveira, Mônica Rodrigues Campos, PNAUM Group, Paulo Sergio Dourado Arrais, Luiz Roberto Ramos, Tatiane da Silva Dal Pizzol, Sotero Serrate Mengue, Mareni Rocha Farias

**Affiliations:** 1 Department of Medicines and Pharmaceutical Policies, National School of Public Health Sergio Arouca, Oswaldo Cruz Foundation, 1480 Rua Leopoldo Bulhões # 624 or 632, Manguinhos, ZC 21041-210, Rio de Janeiro, RJ, Brazil; 2 Pharmacy Department, Health Science School, University of Brasília (UnB), Campus Darcy Ribeiro, Asa Norte, Brasilia, DF, 70910-900, Brazil; 3 Department of Social Medicine, School of Medicine, Postgraduate Program in Epidemiology, Federal University of Pelotas, 1160 Marechal Deodoro Street, Pelotas, RS, 96020-220, Brazil; 4 Department of Social Medicine, School of Medicine, Federal University of Rio Grande do Sul, 2400 Ramiro Barcelos Street, Porto Alegre, RS, 90.035-003, Brazil; 5 Department of Social Sciences, National School of Public Health Sergio Arouca, Oswaldo Cruz Foundation, 1480 Rua Leopoldo Bulhões # 624 or 632, Manguinhos, ZC 21041-210, Rio de Janeiro, RJ, Brazil

**Keywords:** Chronic non-communicable diseases, rational use of medicines, primary health care, household survey

## Abstract

This article aims to describe the inappropriate use of medicines in the Brazilian urban population and to identify associated factors. We conducted a data analysis of a household survey carried out in Brazil in 2013–14. The sampling plan was done by clusters with representativeness of the urban population and large regions of the country, according to gender and age domains. For this analysis, we considered a sample of adults (≥20 years) who reported having chronic non-communicable diseases, medical indication for drug treatment and medicine use (*n* = 12 283). We evaluated the prevalence of inappropriate use in the domains: non-adherence, inappropriate use behaviour and inadequate care with medicines, all verified in the following groups of independent variables: demographic and socio-economic characteristics, health and pharmaceutical care, health status and use of medicines. Crude and adjusted prevalence ratios were obtained using robust Poisson regression. It was found 46.1% of people having at least one behaviour of inappropriate use of medicines. The worst results were found for the domain of inappropriate use behaviour, a situation of 36.6% of the users, which included unauthorized prescriber, inadequate source of information and indication of the medicines by non-authorized prescribers. The best result was found for the lack of medicines care, informed by only 4.6% of users who kept expired drugs at home. The inappropriate use of medicines was associated with gender (female), region of residence (Northeast), not visiting the doctor regularly or visiting more than one doctor, not having free access to medicines and using of five or more medicines. There was a high prevalence of inappropriate use, which was associated with both individual and health system characteristics pointing out the need to set priorities as for health education and public interventions.

**Key Messages**

- About half of people had at least one inappropriate medicines use behaviour.
- The inappropriate use of medicines was associated both with the characteristics of the individuals, treatment, and of the health system.
- Our results point out the need to enforce health education of individuals, families and community regarding to medicines as well as health system strengthening measures.
- This study proposes a synthetic indicator to inappropriate use of medicines, after application in a national household survey data.

## Background

The appropriate use of medicines (AUM) is inalienably linked to access to medicines as an important health system goals, both fundamental to achieve universal access to health care and coverage (Wirtz *et al.*, 2017). When proposing the six building blocks of a health system, the assurance of equitable access to essential medical medicines, vaccines and technologies of assured quality, safety, efficacy and cost-effectiveness is together with the assurance of their scientifically sound and cost-effective use (WHO, 2007).

The medication process involves a wide set of stakeholders from inside, as prescribers, dispensers, care keepers and patients, as well as from outside the health system, as medicines producers and vendors. However, the patients, the ones who really deal with the consequences of medicines use, should be at the core of health system efforts. Household surveys provide a unique opportunity to capture users perspectives (Short Fabic *et al.*, 2012) and can be applied in regards to the practices and experiences related to medicines (Mengue *et al.*, 2016).

The World Health Organization, as well as other broad initiatives, as ‘Medicines Transparency Alliance’ have been investing on medicines use studying methods which are suitable to be applied in low- and middle-income countries, that generally do not count on robust and well-structured information systems on this issue. The most developed approach is the one applied in healthcare facilities (WHO, 2006). It has been valuable to show problems regarding availability of medicines, storage conditions and some issues regarding appropriate use problems perceivable at the health facility level, as adherence of prescribers to standard treatment protocols and the average number of medicines per prescription (WHO, 2009). A household approach also exists, enabling countries to raise relevant information (WHO, 2016).

Nevertheless, there is still the need for indicators able to point out relevant group of problems in order to monitor and guide policymaking addressing the promotion of AUM. As an example, despite the access to medicines being a target among the Millennium Development Goals, there is no indicator to monitor it (Gotham *et al.*, 2016).

Intending to start a monitoring system on pharmaceutical policies, the Brazilian Ministry of Health promoted the conduction of a National Survey on Access, Use and Promotion of Rational Use of Medicines (PNAUM) with two components: evaluation of the dispensing facilities and a household survey.

Both were able to raise interesting data on the access and use of medicines. It was found 94.3% [95% confidence interval (CI): 93.4–95.1] of full access to non-communicable diseases (NCDs) medicines, 30.8% (95%CI: 28.8–33.0) of low adherence prevalence to drug treatment for NCD (Tavares *et al.*, 2016) and 16.1% (95%CI: 15.0–17.5) of self-medication (Arrais *et al.*, 2016).

Nevertheless, a synthetic indicator on the AUM has not been presented until now, and it is considered potentially useful to facilitate its monitoring strategies.

In this article, we aim to describe the inappropriate use of medicines in the Brazilian population at a household level using a synthetic indicator, identifying associated variables.

## Methods

We used data from the National Survey on Access, Use and Promotion of Rational Use of Medicines (PNAUM—*Pesquisa Nacional Sobre Acesso, Utilização e Promoção do Uso Racional de Medicamentos*) a nationwide household survey with a representative sample of the Brazilian urban population at national and administrative regions level. Since the use of medicines varies according to age and gender, the sampling considered a balance of these two variables. The results were adjusted by post-stratification weights considering the low response taxes. The data were collected between September 2013 and February 2014. More details on the PNAUM methods can be found elsewhere (Mengue *et al.*, 2016).

The study population for this analysis was of 12 283 people aged 20 years or more who reported being informed by a physician to have a chronic disease (NCD), reported receiving indication to treat it with medicines and being in use of these medicines.

The inappropriate use of medicines was evaluated considering the report of at least one inappropriate behaviour on the use of medicines. The analysed misbehaviours were classified in three groups as follows: (1) *Non-adherence*, which includes (i) therapy interruption, (ii) missed doses, (iii) reduction of doses and (iv) taking extra doses; (2) *inadequate medicine use behaviour*, which includes (i) medicines indicated by other people besides doctors and dentists, (ii) information about medicines in non-reliable sources and (iii) indication of medicines to other people; and (3) *Inadequate care with medicines*, which considers keeping expired medicines at home.

These indicators were adapted from Mendes *et al.* (2014), who proposed a composite indicator to evaluate the AUM by hypertensive and/or diabetic patients treated in primary healthcare units in the city of Rio de Janeiro. This indicator was constructed from different theoretical constructs, whose information came from different sources: direct observation of the field researchers, interviewees’ reports from dichotomous questions, multiple choice and Likert scale and combined three domains (adherence, auto-medication and adequate behaviour regarding medicines—ask for information only to health professionals, no expired medicines neither damaged medicine package or label, all medicines prescribed by authorized prescribers). More information is available in the published article.

**Figure 1.**
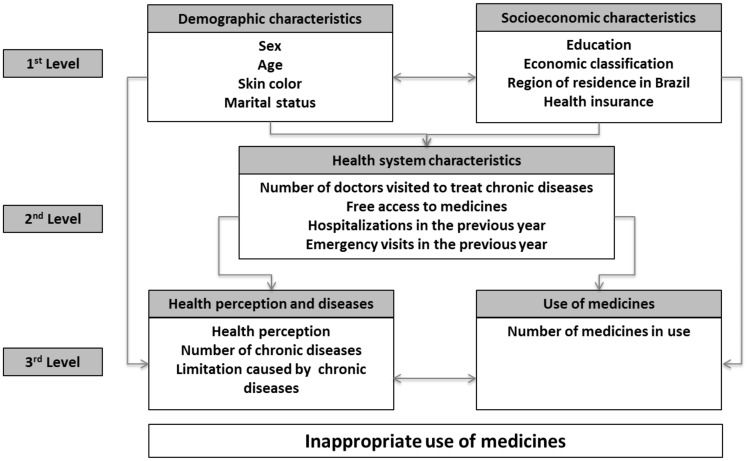
Hierarchical analysis model of the studied factors and its associations to the inappropriate use of medicines. PNAUM, Brazil, 2014. Source: adapted from Tavares *et al.* (2016).

The independent variables under analysis were: sex (male, female); age group (20–39, 40–59, 60 years old or more); skin colour (white, non-white); marital status (married, single); years of formal education (0, 1–8, 8 or more); economic classification (A/B, C, D/E) according to the Brazilian Economic Classification Criterion of the Brazilian Association of Research Companies (CCEB 2013/ABEP—*Associação Brasileira de Empresas de Pesquisa* (http://www.abep.org/); residential geographical region (North; Northeast; Southeast; South, Midwest); health insurance (yes, no); visiting the doctor to treat chronic diseases (no, yes one, yes more than one); free access to the medicines to treat chronic diseases (all, some, none); hospitalization in the previous year (no, once, two or more times); emergency care visits in the previous year (no, once, two or more times); health perception (very good, good, regular, bad, very bad); the number of chronic diseases (1, 2, 3 or more); limitation caused by chronic diseases (yes, no); the number of medicines in use (1, 2, 3–4, 5 or more) (Figure 1).

An exploratory descriptive analysis was conducted; in addition to the prevalence estimates, 95% confidence intervals (95%CI) were calculated and the Pearson’s chi-square test was applied to assess the statistical significance of the differences among the groups, considering a 5% level of significance.

The Poisson regression model was used to estimate the crude and adjusted prevalence ratios (PR), and the 95%CI, to the aggregate as well as to single component’s outcomes. We attempted to control possible confounding factors in the multivariate analysis, using a hierarchical analysis model (Figure 1). Variables with *P* < 0.20 were included in the multiple model and a significance level of 5% was adopted to maintain the variables in the model, along with a backward selection of the variables. The statistical significance of the PR obtained from the Poisson regression models was evaluated using the Wald test.

The analyses were conducted with Stata V.12, using the set of svy commands to analyse complex samples and guaranteed the required weighting by contemplating the characteristics of the sample design that used different sampling fractions and post-stratification weights to correct the response rate flaw.

The study’s design was submitted and approved by the National Ethical Research Committee (CONEP). All the interviews were conducted after the respondent or his/her legal guardian had signed an informed consent term.

## Results

Regarding the socio-economic-demographic characteristics, the weighted estimates indicated that half of the respondents self-declared themselves as white skin colour, there was a higher prevalence of women (64.8%), adults aged between 40 and 59 years (42.7%), married (61.4%), with time spent in education between 1 and 8 years (42.8%), 54.6% belonging to economic class C, 51.4% residing in the Southeast region and 71.9% having no health insurance. Regarding the health services utilization, the majority of the respondents reported visiting the doctor to treat chronic diseases (62.6%), having free access to the medicines (46.7%), not being hospitalized in the previous year (89.2%) or visiting the emergency in the previous year (76.4%). Regarding health perception and diseases, most of the respondents said their health was good (45.3%) or regular (41.2%), 45.6% reported one chronic disease and 84.2% declared having limitations caused by their chronic diseases. Considering the use of medicines, the majority was in use of three or more medicines at the time of the interview (54.4%) (Table 1).

**Table 1:** Sample distribution and prevalence of inappropriate use of medicines according to demographic, socio-economic and health system characteristics, health perception, diseases and use of medicines

Variables	Sample distribution		Proportion of inappropriate medicine use	
	%	95%CI	%	95%CI
Demographic characteristics				
Sex			*<0.001* ^a^	
Male	35.2	33.9–36.6	39.4	36.1–42.9
Female	64.8	63.4–66.1	49.8	46.8–52.7
Age group (years)			*<0.001* ^a^	
20–39	16.3	14.8–17.9	57.5	52.3–62.5
40–59	42.7	41.1–44.3	46.3	43.0–49.6
≥60	41.0	39.2–42.8	41.5	38.5–44.5
Skin color^b^			*0.001* ^a^	
White	50.1	47.3–52.9	43.1	39.9–46.3
Non-white	49.9	47.1–52.7	48.4	45.0–51.9
Marital status^b^			*0.021* ^a^	
Married	61.4	59.8–62.9	44.8	41.6–48.0
Single	38.6	37.1–40.2	48.4	45.1–51.6
Socio-economic characteristics				
Education (years)^b^			*0.153* ^a^	
0	15.2	13.9–16.6	47.0	42.9–51.2
1–8	42.8	40.9–44.7	44.4	41.0–47.9
≥8	42.0	40.1–43.9	47.6	44.2–51.0
Economic classification^c^			*0.636* ^a^	
A/B	24.5	22.2–26.9	47.5	43.0–52.1
C	54.6	52.7–56.5	45.5	42.3–48.7
D/E	20.9	19.1–22.8	46.3	42.0–50.7
Region			*<0.001* ^a^	
North	4.4	3.4–5.6	44.7	39.0–50.4
Northeast	21.2	17.3–25.7	61.3	57.5–64.9
Southeast	51.4	45.5–57.3	41.9	37.1–47.0
South	15.4	12.4–18.9	38.3	35.2–41.5
Midwest	7.7	6.0–9.8	49.0	45.3–52.7
Health insurance (private)^b^			*0.904* ^a^	
Yes	28.1	25.7–30.7	46.3	42.2–50.6
No	71.9	69.3–74.3	46.1	43.0–49.2
Health service utilization characteristics				
Visit the doctor to treat chronic diseases^b^			*<0.001* ^a^	
No	7.9	6.9–9.0	66.7	61, 1–71, 8
Yes, one	62.6	60.5–64.6	41.7	38, 5–44, 9
Yes, more than one	29.5	27.9–31.2	51.8	48, 4–55, 2
Free access to the medicines to treat chronic diseases^b^			*<0.001* ^a^	
All	46.7	44.3–49.1	41.4	38, 0–45, 0
Some	20.2	19.0–21.4	49.3	45, 1–53, 5
None	33.1	31.0–35.3	49.1	45, 9–52, 2
Hospitalized in the previous year^b^			*0.180* ^a^	
No	89.2	88.3–90.0	45.8	42, 9–48, 8
Once	8.2	7.5–9.0	48.0	43, 1–53, 0
Two or more times	2.7	2.2–3.1	53.1	45, 1–61, 0
Emergency visits in the previous year^b^			*<0.001* ^a^	
No	76.4	74.6–78.0	43.1	40, 1–46, 1
Once	15.4	14.3–16.5	54.5	50.3–58.5
Two or more times	8.3	7.3–9.3	59.2	54.2–63.9
Health perception and diseases				
Health perception			*<0.001* ^a^	
Very good	5.1	4.4–5.8	42.4	34.7–50.4
Good	45.3	43.3–47.2	41.0	37.6–44.4
Regular	41.2	39.5–12.9	50.3	47.0–53.6
Bad	6.3	5.6–7.0	54.2	49.4–59.0
Very bad	2.2	1.9–2.6	59.1	50.9–66.9
Number of chronic diseases			*<0.001* ^a^	
1	45.6	43.7–47.5	41.1	37.8–44.5
2	27.1	25.9–28.2	47.5	44.4–50.7
3 or more	27.3	25.7–29.0	53.1	49.3–56.9
Limitation caused by chronic diseases^b^			*0.036* ^a^	
Yes	84.2	83.0–85.4	47.0	44.4–49.9
No	15.8	14.6–17.0	42.8	38.4–47.2
Use of medicines				
Number of medicines in use			*<0.001* ^a^	
1	21.6	20.3–22.9	34.8	31.4–38.3
2	24.0	20.3–22.9	42.7	38.6–47.0
3–4	30.9	293–32.3	48.8	45.5–52.2
5 or more	23.5	22.0–25.0	56.5	52.6–60.3
Total			46.1	43.3–49.0

Percentages adjusted by sample weights and post-stratification according to age and gender. Pnaum, Brazil, 2014 (*N* = 12 283).

a Pearson’s chi-square test.

b Variable with missing values.

c The Economic Classification variable is according to the 2013 Brazilian Economic Classification Criterion of the Brazilian Association of Research Companies (www.abep.org).

The prevalence of inappropriate use of medicines was 46.1%. From these, 55.3% reported only one misbehaviour, 27.8% reported two and 16.9% three or more. With reference to the inadequate use domains, the highest prevalence was found on the non-adherence (31.9%) group, with greater and smallest highlights in the proportion of people that reduced medication doses (19.8%) and the proportion of people who took an extra dose or more medication than prescribed (7.4%). Following, the prevalence found for the inadequate medicine use behaviour domain was 36.6%, in which the highest and smallest prevalence was found in the indicators that considered the proportion of people who had their medicines indicated by other people besides doctors and (34.3%) and the indication of medicines to other people (7.4%). Finally, the smallest prevalence, meaning the best result for the inadequate use of medicines, was found for the domain that considers the inadequate care with medicines, with 4.6% of the respondents with expired medicines at home (Table 2).

**Table 2:** Inappropriate use of medicines domains and its indicators of analysis along with its prevalence in the study population

Indicator	Origin variable	%	LCEMP^a^
IMU 1 Non-adherence At least one inadequate behaviour from the list above (UAM1)		31.9	
*IMU 1.1* Proportion of people who interrupted therapy	Remained without medicines for the treatment of your chronic diseases in the last 30 days?	9.9	Underuse
*IMU 1.2* Proportion of people who reported any missed doses for chronic treatments	Did you forget any dose in the last week or month?	8.5	Underuse
*IMU 1.3* Proportion of people who reduced medication doses	Do you reduce medication dose when the disease is controlled, when the medicines makes you fill bad, when you want it to last longer or when it is expensive?	19.8	Underuse
*IMU 1.4* Proportion of people who took any extra dose or more medication than prescribed	Do you increase medication dose when you want to start a stronger treatment, when you notice improvement or when you feel worse?	7.4	Overuse
IMU 2 Inadequate medicine use behaviour At least one inadequate behaviour from the list above (UAM2)		36.6	
*IMU 2.1* Proportion of people who had their medicines indicated by other people besides doctors and dentists	Who indicated this medicine?	34.3	Misuse
*IMU 2.2* Proportion of people who sought for information about medicines in non-reliable sources such as relatives or other people and services not related to health	When you have any questions about using medicines, where or with whom you usually seek for information?	5.9	Misuse
*IMU 2.3*—Indication of medicines to other people	Did you indicate this medicine to other people?	17.7	Misuse
IMU 3 inadequate care with medicines At least one expired medicine (UAM3)		4.6	
*IMU 3.1* Proportion of people with at least one expired medicine	Expire date of the medicine	4.6	Misuse

PNAUM, Brazil, 2014 (*N* = 12 283).

a The Lancet Commission on Essential Medicines Policies.

The bivariate analysis shows a higher inappropriate use of medicines in women (49.8%), aged between 20 and 39 years (57.5%), non-white skin colour (48.4%), single (48.4%), residing in the Northeast region of Brazil (61.3%), who didn’t visit the doctor to treat their chronic diseases (66.7%), with free access to medicines (49.3%) and who reported having limitations caused by chronic diseases (47%). The number of emergency visits in the previous year, health perception, number of chronic diseases and use of medicines showed proportional gradients to the inappropriate use of medicines; the highest prevalence were: two or more emergency visits in the previous year (59.2%) very bad health perception (59.1%), three or more chronic diseases (53.1%) and five or more medicines in use (56.5%). The number of hospitalizations in the previous year, the economic classification, education and health insurance didn’t have a statistically significant relation to the inappropriate use of medicines (Table 1).

In regards to the multivariable analysis, performed through Poisson regression, the inappropriate use of medicines profile was found as follows: women (PR = 1.21), ages between 20 and 39 years (PR = 1.44), residing in the Northeast region of Brazil (PR = 1.19), who reported not visiting the doctor to treat chronic diseases (PR = 1.30; 95%CI: 1.18–1.44), with no free access to medicines (PR = 1.14), with two or more emergency visits in the previous year (PR = 1.12) and five or more medicines users (PR = 2.0). Besides that, an important variation between the crude and adjusted PR was found in the following variables: ‘Northeast region’ (crude PR = 1.37 vs adjusted PR = 1.19), ‘two or more times emergency visits in the previous year’ (crude PR = 1.37 vs adjusted PR = 1.12) and ‘5 or more medicines in use’ (crude PR = 1.62 vs adjusted PR = 2.00) (Table 3). The model applied to the aggregate inappropriate use of medicines outcome translates the behaviour of the single components to all independent variables, implying a good consistency of the contribution of components to the composite indicator.

**Table 3:** Crude and adjusted PR on the inappropriate use of medicines and its components (non-adherence, inadequate medicine use behaviour and inadequate care with medicines) according to demographic, socio-economic and health system characteristics, health perception, diseases and use of medicines

Variables	Inappropriate use of medicines (general)				Non-adherence		Inadequate medicine use behaviour		Inadequate care with medicines	
	Crude PR		Adjusted PR^a^		Adjusted PR^a^		Adjusted PR^a^		Adjusted PR^a^	
	RP	CI95%	RP	CI95%	RP	CI95%	RP	CI95%	RP	CI95%
Demographic characteristics										
Sex	*<0.001* ^b^		*<0.001* ^b^		*<0.001* ^b^		*<0.001* ^b^			
Male	Ref.		Ref.		Ref.		Ref.			
Female	1.26	1.18–1.35	1.21	1, 14–1, 29	1.18	1.09–1.29	1.37	1.21–1.55		
Age group (years)	*<0.001* ^b^		*<0.001* ^b^		*<0.001* ^b^		*<0.001* ^b^			
20–39	1.38	1.26–1.52	1.44	1, 31–1, 58	1.74	1.53–1.98	1.56	1.30–1.88		
40–59	1.12	1.05–1.19	1.14	1, 07–1, 21	1.19	1.09–1.30	1.18	1.05–1.33		
≥60	Ref.		Ref.		Ref.		Ref.			
Skin colour^c^	*0.001* ^b^									
White	Ref.									
Non-white	1.13	1.05–1.21								
Marital status^c^	*0.208* ^b^									
Married	Ref.									
Single	1.08	1.01–1.15								
Socio-economic characteristics										
Education (years)^c^	*0.157* ^b^									
0	Ref.									
1–8	0.98	0.90–1.09								
≥8	0.93	0.87–1.01								
Economic classification^d^	*0.647* ^b^						*0.039* ^b^			
A/B	Ref.						Ref.			
C	0.96	0.87–1.06					0.89	0.77–1.04		
D/E	0.97	0.86–1.10					0.80	0.67–0.95		
Region	*<0.001* ^b^		*<0.001* ^b^		*<0.001* ^b^		*<0.001* ^b^			
North	Ref.		Ref.		Ref.		Ref.			
Northeast	1.37	1.19–1.58	1.19	1.05–1.35	1.24	1.05–1.45	1.12	0.93–1.34		
Southeast	0.94	0.79–1.12	0.88	0.75–1.02	0.88	0.73–1.06	0.76	0.62–0.93		
South	0.86	0.74–1.0	0.83	0.72–0.95	0.82	0.69–0.97	0.69	0.55–0.85		
Midwest	1.10	0.95–1.27	0.99	0.87–1.13	0.98	0.83–1.16	0.79	0.65–0.96		
Health insurance^c^	*0.904* ^b^									
Yes	1.01	0.92–1.10								
No	Ref.									
Health system characteristics										
Visit the doctor to treat chronic diseases^c^	*<0.001* ^b^		*<0.001*		*<0.001* ^b^		*<0.001* ^b^		*0.005* ^b^	
No	1, 29	1, 17–1, 41	1, 31	1, 19–1, 45	1.26	1.11–1.43	1.62	1.36–1.92	1.88	1.23–2.89
Yes, one	0, 80	0, 75–0, 86	0, 90	0, 85–0, 97	0.80	0.73–0.88	0.97	0.86–1.08	1.00	0.73–1.37
Yes, more than one	Ref.		Ref.		Ref.		Ref.		Ref.	
Free access to the medicines to treat chronic diseases^c^	*0.011* ^b^		*0.001*				*<0.001* ^b^			
All	Ref.		Ref.				Ref.			
Some	1.19	1.10–1.29	0.98	0.91–1.05			0.85	0.74–0.99		
None	1.18	1.09–1.29	1.14	1.05–1.23			1.27	1.12–1.43		
Hospitalized in the previous year^c^	*0.108* ^b^						*0.014* ^b^			
No	Ref.						Ref.			
Once	1.05	0.95–1.16					0.80	0.68–0.96		
Two or more times	1.16	1.0–1.35					0.78	0.59–1.05		
Emergency visits in the previous year^c^	*<0.001* ^b^		*0.012*		*<0.001* ^b^					
No	Ref.		Ref.		Ref.					
Once	1.26	1.17–1.36	1.09	1.01–1.17	1.14	1.04–1.26				
Two or more times	1.37	1.25–1.50	1.12	1.03–1.22	1.21	1.08–1.36				
Health perception and diseases										
Health perception	*<0.001* ^b^								*0.003* ^b^	
Very good	Ref.								Ref.	
Good	0.97	0.81–1.16							0.55	0.22–1.41
Regular	1.19	0.99–1.42							0.70	0.27–1.78
Bad	1.28	1.05–1.56							0.96	0.37–2.51
Very bad	1.40	1.16–1.68							1.58	0.53–4.71
Number of chronic diseases	*<0.001* ^b^				*<0.001* ^b^		*0.017* ^b^			
1	Ref.				Ref.		Ref.			
2	1.16	1.08–1.24			1.25	1.13–1.38	0.84	0.73–0.97		
3 or more	1.29	1.19–1.40			1.42	1.29–1.56	0.81	0.68–0.95		
Limitation caused by chronic diseases^c^	*0.042* ^b^									
Yes	Ref.									
No	0.91	0.83–1.0								
Use of medicines										
Number of medicines in use	*<0.001* ^b^		*<0.001* ^b^						*<0.001* ^b^	
1	Ref.		Ref.				Ref.		Ref.	
2	1.23	1.11–1.37	1.39	1.23–1.57			3.61	2.66–4.92	1.69	0.86–3.32
3–4	1.40	1.29–1.53	1.64	1.47–1.82			5.95	4.50–7.87	3.91	2.07–7.41
5 or more	1.62	1.46–1.80	2.00	1.77–2.26			8.14	6.0–11.05	7.33	4.02–13.40

PR adjusted by sample weights and post-stratification according to age and gender. Pnaum, Brazil, 2014. (*N* = 12 283).

a Variable selection based on backward deletion (variables with *P* < 0.20 were included in multiple model and a significance level of 5% was adopted to maintain the variables in the model).

b Wald’s test.

c Variable with missing values.

d The Economic Classification variable is according to the 2013 Brazilian Economic Classification Criterion of the Brazilian Association of Research Companies (www.abep.org).

Despite statistical significance of the economic classification variable regarding inadequate medicine use behaviour component, this was not translated to the aggregate indicators, neither to the other components (Table 3).

From the analysis of the separate models for each component (Table 3), we found a more expressive effect in the single components but more attenuated in the aggregate indicator for the variable ‘Visit the doctor to treat chronic diseases’ where adjusted PR is 1.3 for the aggregate outcome and reaches 1.9 for component 3 (drug use with expired date). On the other hand, the adjusted PR of 0.8 seems to be protective in relation to non-adherence for those who made at least one visit compared with those who did not. No significant result for the adjusted PR of the variable number of chronic diseases to the aggregate indicator was found, but a positive to the non-adherence and protective effect to the inadequate behaviour was estimated (Table 3).

## Discussion

The Lancet Commission on Essential Medicines proposes a taxonomy for the inappropriate use of medicines classified in four categories: unnecessary medicine use (overuse); incorrect medicines use (misuse); failure to use needed medicines (underuse); and unnecessary use of highly priced medicines (Wirtz *et al.*, 2017). In this current article, we were able to approach the first three categories using a secondary database from a household survey through a synthetic indicator, potentially contributing to the possibility of include this approach in a monitoring system.

There is an effort in Brazil to build longitudinal databases on medicines use. From the two main medicines provision mechanisms in place, ‘*Farmacia Popular* Program’ counts on a well-structured database, but it is not that easy to be accessed by researchers (Luiza *et al.*, 2018) and there is an on-going, but still incomplete, effort to implement ‘Horus’ (Costa and Nascimento, 2012), a pharmacy management software which offers data on medicines provision in public health facilities. Then, PNAUM, was the first study able to offer a broad picture on access to and use of medicines at national and regional level (Bertoldi *et al.*, 2016).

In PNAUM, we already achieved a synthetic indicator on access to medicines to treat NCDs that have been increasingly used in the country. Despite approaching different aspects of inappropriate use of medicines in previous publications, we were still missing an equivalent synthetic indicator to it.

Mendes *et al.* (2014) proposed a composite indicator to estimate the AUM combining three domains (adherence, no auto-medication and adequate behaviour regarding medicines—ask for information only to health professionals, no expired medicines neither damaged medicine package or label, all medicines prescribed by authorized prescribers) and applied in data from a household survey conducted in Rio de Janeiro (about 6.3 million inhabitants). In this article, we adapted their proposal to have a picture of the whole country.

The distribution of the respondents’ characteristics variables expressed challenges faced on healthcare utilization behaviour and access, country inequities as well as some health system achievements at that time to mitigate inequity. The sample comprises people reporting the diagnosis of NCD (access to healthcare), medicine prescription and medicine use (access to medicines). The data suggest, as e.g. a highest proportion of women which is linked to the fact that they are the most frequent health services users (Levorato *et al.*, 2014). In the same way, the majority being from the Southeast region points out inequalities in health services distribution (Viacava and Bellido, 2016), and in consequence, of diagnosis capacity. The distribution pattern of other variables as skin colour and private health insurance coverage show improvements in equity.

The results showed around half people presenting at least one inappropriate medicines use behaviour, non-adherence and inadequate use were the highest ones. Non-adherence to medication was shown to be one of the main components of the inadequate use indicator analysed. The low adherence to the drug treatment for chronic diseases in Brazil is relevant, with regional and demographic differences and related to the healthcare of the patients pointing to the need of monitoring strategies in the health services (Tavares *et al.*, 2016).

Most of the problems in non-adherence domain were linked to dosage reduction/interruption of treatment, which may be linked to access to medicines problem. Indeed, the literature reports controversial results on access to medicines. Despite the prevalence of general access to NCD medicines is high, from 82.5% (Stopa *et al.*, 2017) to 94.3% (Oliveira *et al.*, 2016), it varies mainly in regards to the disease, the number of disease conditions and the user’s living country or region (Oliveira *et al.*, 2016).

We found a high prevalence of people using non-formal sources for medicines prescription (in Brazil, legal prescribers are physicians, dentists and nurses, these last in some specific cases). These prescription sources include a huge varied of possibilities as family, neighbours, private pharmacy clerks (there is no legal requirement for minimal training in Brazil) and internet. Despite the problems linked to each of these, the last calls for attention, it is an increasing source of health self-care, including self-medication, with a big variety on quality of information among websites. Indeed, Gondim *et al.* (2012) documented the generally poor quality of information about medicines on Brazilian websites.

The adjusted prevalence rate did not show an association of inappropriate use of medicines and economic classification or education, suggesting that equity policies applied around the time of data collection could have been successful (Luiza *et al.*, 2016).

Independent variables found to be linked to the inappropriate use of medicines offer information on where to prioritize attention and efforts to mitigate this problem. As an example, demographic characteristics indicate whose user profile should be prioritized in educational campaigns as well as professional continuing education in order they are aware when managing these people’s health. The Northeast is clearly a region where efforts should be intensified. Also, it was made clear how far healthcare use characteristics, as the frequency of ambulatory visit and use of emergency care, showed to be linked to the problem of inappropriate use of medicines. The results demonstrate the importance of the physician–patient relationship, the so-called ‘joint empowerment’ approach to promoting adherence and appropriate use of medications in these scenarios (Náfrádi *et al.*, 2017).

As main limitations of our study, we had information being self-reported by lay people. On the one hand, we could capture their perspective. On the other, some inaccuracies may have been present. Additionally, despite the data were collected by independently trained interviewers, since it was presented as a Ministry of Health study, some inappropriate behaviour may have been under-reported. The presence of family members during the interview may also have affected the reliable reporting of some behaviours. It is important to consider that, as we used a secondary database, the sample was not designed specifically for the analysis we performed in this article and variables used here were collected in different parts of the questionnaire. However, we see as positive the possibility of using data from a nationwide survey to study AUM, which can stimulate others to do the same. Also, although PNAUM tried to work with a narrow recall period, 1 month in the case of NCDs, this may have hampered some recall of information by the respondents.

Finally, a high prevalence of inappropriate use of medicines was shown, mainly in the non-adherence and inadequate medicines use behaviour domains, which was associated with both the characteristics of the individuals, treatment and the health system. Individuals’ characteristics are not modified by interventions, but our finding could support priorities to health education regarding medicines use and definition of target populations. Also, it is clear that is important to assure regular visit to physicians. For example, overmedication was associated with inappropriate use of medicines, suggesting the need to prioritize medicines counselling strategies. We would also like point out that the inappropriate use of medicines brings consequences to the health systems, like a higher use of emergency care. We hope to have contributed to the inappropriate use of medicines monitoring system.

## Ethical approval

PNAUM was approved by Comissão Nacional de ȴica em Pesquisa (National Research Ethics Commission – Protocol 18947013.6.0000.0008). All interviews were conducted after the respondents or their legal representatives (in the case of incapable persons) had read and signed the informed consent, with assurance of confidentiality and anonymity.
